# Other PHAC publications

**DOI:** 10.24095/hpcdp.44.1.06

**Published:** 2024-01

**Researchers from the Public Health Agency of Canada also contribute to work published in other journals and books. Look for the following articles published in 2023:**

Akl EA, Cuker A, Mustafa RA, Nieuwlaat R, **Stevens A**, et al. Prospective collaborative recommendation development: a novel model for more timely and trustworthy guidelines. J Clin Epidemiol. 2023;162:156-9. https://doi.org/10.1016/j.jclinepi.2023.08.015 

Bird MJ, Li G, MacNeil A, **Jiang Y, de Groh M**, et al. Pandemic-induced depression among older adults with a history of cancer during the COVID-19 pandemic: findings from the Canadian Longitudinal Study on Aging. Cancer Manag Res. 2023;15:937-55. https://doi.org/10.2147/CMAR.S421675 

Biswas A, Chen C, Dobson KG, **Prince SA**, et al. Identifying the sociodemographic and work-related factors related to workers’ daily physical activity using a decision tree approach. BMC Public Health. 2023;23(1):1853. https://doi.org/10.1186/s12889-023-16747-9

Fuller D, Shareck M, Sersli S, […] **Lang JJ, Wolfe Phillips E**. Common measures of green and blue space for built environment, health equity and intervention research: a scoping review. Cities Health. 2023;7(6):1118-25. https://doi.org/10.1080/23748834.2023.2260134

Paprica PA, Crichlow M, Maillet DC, […] **Dostmohammad S**, et al. Essential requirements for the governance and management of data trusts, data repositories, and other data collaborations. Int J Popul Data Sci. 2023;8(4):01. https://doi.org10.23889/ijpds.v8i4.2142

Sandhu HS, **Otterman V**, Tjaden L, **Shephard R**, et al. The governance of core competencies for public health: a rapid review of the literature. Public Health Rev. 2023;44:1606110. https://doi.org/10.3389/phrs.2023.1606110

Shareck M, Fuller D, Sersli S, […], **Lang JJ, Wolfe Phillips E**. Measuring walkability and bikeability for health equity and intervention research: a scoping review. Cities Health. 2023;7(6):1108-17. https://doi.org/10.1080/23748834.2023.2260133

Taunque A, Li G, Macneil A, […] **Jiang Y, de Groh M.** Breathless and blue in the Canadian Longitudinal Study on Aging: incident and recurrent depression among older adults with COPD during the COVID-19 pandemic. Int J Chron Obstruct Pulmon Dis. 2023;18:1975-93. https://doi.org/10.2147/COPD.S417218

